# Essentials of Chemistry. Organic and Inorganic

**Published:** 1881-04

**Authors:** 


*Essentials of Chemistry.*—Organic and inorganic. For the use of students in medicine. By R. A. Witthaus, A. M., M. D. Prof. of Chemistry University of Vermont. Wm. Wood & Co., New York. 1880.

A pocket volume of about 250 pages, neatly arranged with concise questions and answers. The author has condensed a great deal of information into a little space, and for a reference book this is an excellent one indeed. It is not a manual of analytical chemistry, and contains " only such analytical process as the physician may reasonably expect to make use of in his daily practice." Physiological chemistry occupies a respectable space; and the modern system of notation is adopted. The weights and measures are given in the metric system, and temperatures in the contigrade scale. As a handy reference book we feel sure it will find a welcome. H.

